# Ovarian stiffness increases with age in the mammalian ovary and depends on collagen and hyaluronan matrices

**DOI:** 10.1111/acel.13259

**Published:** 2020-10-20

**Authors:** Farners Amargant, Sharrón L. Manuel, Qing Tu, Wendena S. Parkes, Felipe Rivas, Luhan T. Zhou, Jennifer E. Rowley, Cecilia E. Villanueva, Jessica E. Hornick, Gajendra S. Shekhawat, Jian‐Jun Wei, Mary Ellen Pavone, Adam R. Hall, Michele T. Pritchard, Francesca E. Duncan

**Affiliations:** ^1^ Department of Obstetrics and Gynecology, Feinberg School of Medicine Northwestern University Chicago IL USA; ^2^ Northwestern University Atomic and Nanoscale Characterization Experimental (NUANCE) Center, Northwestern University Evanston IL USA; ^3^ Department of Pharmacology, Toxicology and Therapeutics University of Kansas Medical Cente Kansas City KS USA; ^4^ Virginia Tech‐Wake Forest University School of Biomedical Engineering and Sciences Wake Forest School of Medicine Winston‐Salem NC USA; ^5^ Biological Imaging Facility (BIF) Northwestern University Evanston IL USA; ^6^ Department of Pathology, Feinberg School of Medicine, Northwestern University Chicago IL USA; ^7^ Present address: Department of Obstetrics and Gynecology Texas Tech Health Sciences Center El Paso TX USA; ^8^ Present address: Department of Materials Science and Engineering Texas A&M University College Station TX USA

**Keywords:** Biomechanics, extracellular matrix, fibrosis, hyaluronan synthase, hyaluronidase, reproduction

## Abstract

Fibrosis is a hallmark of aging tissues which often leads to altered architecture and function. The ovary is the first organ to show overt signs of aging, including increased fibrosis in the ovarian stroma. How this fibrosis affects ovarian biomechanics and the underlying mechanisms are unknown. Using instrumental indentation, we demonstrated a quantitative increase in ovarian stiffness, as evidenced by an increase in Young's modulus, when comparing ovaries from reproductively young (6–12 weeks) and old (14–17 months) mice. This ovarian stiffness was dependent on collagen because *ex vivo* enzyme‐mediated collagen depletion in ovaries from reproductively old mice restored their collagen content and biomechanical properties to those of young controls. In addition to collagen, we also investigated the role of hyaluronan (HA) in regulating ovarian stiffness. HA is an extracellular matrix glycosaminoglycan that maintains tissue homeostasis, and its loss can change the biomechanical properties of tissues. The total HA content in the ovarian stroma decreased with age, and this was associated with increased hyaluronidase (*Hyal1*) and decreased hyaluronan synthase (*Has3*) expression. These gene expression differences were not accompanied by changes in ovarian HA molecular mass distribution. Furthermore, ovaries from mice deficient in HAS3 were stiffer compared to age‐matched WT mice. Our results demonstrate that the ovary becomes stiffer with age and that both collagen and HA matrices are contributing mechanisms regulating ovarian biomechanics. Importantly, the age‐associated increase in collagen and decrease in HA are conserved in the human ovary and may impact follicle development and oocyte quality.

## Graphical abstract

Advanced reproductive age is associated with a quantitative increase in ovarian tissue stiffness. Both collagen and hyaluronan (HA) matrices regulate the biomechanical properties of the ovary, and with age, there is an increase in ovarian collagen and a decrease in HA (without a change in HA polydispersity). These age‐dependent changes in extracellular matrix molecules are conserved in mouse and human. The increased stiffness of the aging ovary likely influences gamete quantity and quality and may be an important therapeutic target to extend reproductive longevity.

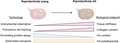

## 1 INTRODUCTION

The female reproductive system is the first to age in the human body, resulting in decreased fertility and also hormonal dysfunction which can impact overall health (Broekmans et al., 2009). Female reproductive aging is a broad health concern as more women are delaying childbearing, resulting in infertility and higher incidences of miscarriages, and birth defects (Duncan et al., 2018). Moreover, lifespan is increasing considerably due to medical interventions while the age of menopause is relatively constant at the age of ~50 years in developed countries (Gold, 2011). Thus, more women will live longer in a period of suboptimal endocrine function. Therefore, there is an urgent need to understand the physiologic and cellular mechanisms that underlie aging of the female reproductive system.

With age, there is a decrease in oocyte quantity and quality (Broekmans et al., 2009). Oocytes develop within a follicle unit surrounded by a complex ovarian stroma, consisting of a variety of cell types and extracellular matrix (ECM) components (Tingen et al., 2011; Wang et al., 2020). The ECM forms a macromolecular network which provides physical support and induces biochemical signaling pathways in cells to maintain or modify tissue morphogenesis, differentiation, homeostatic status, and mechanical properties (Monslow et al., 2015; Tingen et al., 2011). However, excessive ECM accumulation can negatively impact tissue function. For example, fibrosis, characterized by collagen I and III accumulation and organ dysfunction, is a common hallmark of several aging tissues, including the lung and heart (Biernacka & Frangogiannis, 2011; Gimenez et al., 2017). Like other organs, the mammalian ovary becomes increasingly fibrotic with age, and this phenomenon is conserved from mouse to human (Briley et al., 2016; McCloskey et al., 2020). However, the ovary is unique in that it is the first to age, so it represents a critical model to investigate the cellular mechanisms underlying fibrosis, to identify novel therapeutic targets, and to screen and test potential anti‐fibrotic agents.

Fibrosis is typically associated with increased tissue stiffness. For example, dermal stiffness correlates with collagen deposition and fibrillar structure (Achterberg et al., 2014), and collagen deposition also increases matrix stiffness in the liver (Chen et al., 2019). This matrix rigidity is sensed by surrounding cells and results in gene expression, inflammatory response, and cell cycle changes (Lampi & Reinhart‐King, 2018). In addition to collagen, other ECM components can regulate tissue micromechanical properties. For example, hyaluronan (HA) is a ubiquitous glycosaminoglycan which, in its native form, binds to water molecules and promotes tissue hydration, micromechanically soft environments, and tissue homeostasis (Monslow et al., 2015; Toole, 2004). HA is synthesized by hyaluronan synthases as a high‐molecular weight (HMW) polymer of repeating disaccharide units forming a linear polysaccharide chain (Monslow et al., 2015). Conversely HA is fragmented into low‐molecular weight (LMW) HA by the activity of hyaluronidases and reactive oxygen species (ROS) limiting its ability to create mechanically soft matrices (Monslow et al., 2015; Toole, 2004).

Given the links between fibrosis, tissue stiffness, and ECM components, we hypothesized that reproductive aging is associated with increased ovarian stiffness precipitated by changes in ovarian stromal ECM composition, namely collagen and HA matrices. Using instrumental indentation and a physiologic aging mouse model, we demonstrated that there is a quantitative increase in ovarian stiffness with advanced reproductive age, which is due in part to increased collagen in the ovarian stroma. Moreover, there is an age‐dependent decrease in ovarian stromal HA which is associated with dysregulation of HA synthase and hyaluronidase expression but not with changes in HA molecular mass. Ovaries from a Has3 genetic loss‐of‐function model were stiffer than wild‐type (WT) controls, suggesting a role of the HA matrix in regulating ovarian stiffness. These findings are the first to provide a quantitative biomechanical metric of ovarian aging and to demonstrate the critical role of collagen and HA matrices in regulating ovarian tissue stiffness. Importantly, the age‐associated increase in collagen and decrease in HA are conserved in the human ovary, underscoring the important translational potential of these findings.

## 2 RESULTS

### 2.1 Age‐associated increase in ovarian stiffness is collagen‐dependent

**Figure 1:**
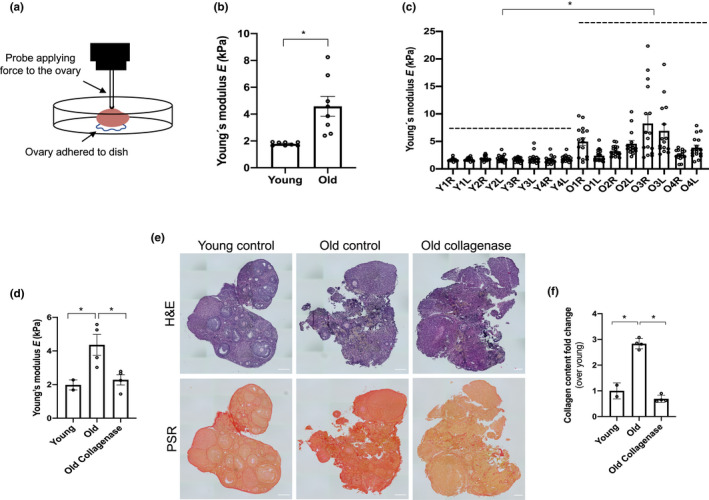
Ovarian stiffness increases with advanced reproductive age and depends in part on the collagen matrix. (a) Schematic of the indentation method. (b‐c) The graphs show ovarian stiffness (Young's modulus, *E*) in all the ovaries combined (b) or (c) in individual ovaries (Y: young, O: old, R: right, L: left). N = 8 ovaries from reproductively young mice, or N = 8 ovaries from reproductively old mice. (d) Graph showing the stiffness (Young's modulus, *E*) of reproductively young (N = 2), reproductively old (control; N = 4), and reproductively old ovaries treated with collagenase (N = 4). (e) Images of post‐indented ovaries fixed and stained with H&E and PSR. Scale bar=100 μm. (f) PSR‐positive area represented as fold change over young (in indented reproductively young and old control ovaries and collagenase treated reproductively old ovaries). Asterisks indicate significant differences (*p* < 0.05)

Aging can influence tissue biomechanics, so to determine whether aging affects the quantitative stiffness of the ovary, we performed instrumental indentation (Figure 1a). This method is used to determine the amount of force required to indent a tissue a given distance, which is a readout of tissue stiffness (Gautier et al., 2015). It took 2.5‐fold more force to indent ovaries from reproductively old mice compared to young (young 1.79 ± 0.08 kPa; old 4.56 ± 2.03 kPa; *p* < 0.05), demonstrating increased ovarian stiffness with age (Figure 1b). When the data were analyzed on an individual ovary basis, ovaries from reproductively young mice were more uniform, whereas there was a higher degree of variability in stiffness measurements in ovaries from reproductively old mice even within the same ovary (Figure 1c).

To explore the potential mechanisms responsible for the age‐associated increase in ovarian stiffness, we focused on the ECM. We previously demonstrated that collagen increases with age in the ovary, and we further validated this histologically in our current samples (*p* < 0.05; Figure S1a‐b) (Briley et al., 2016). To determine whether the increase in ovarian stiffness was dependent on collagen, we decreased collagen content of ovaries, *ex vivo*, using a brief collagenase treatment. Similar approaches have been used to modulate collagen in embryonic and neonatal ovaries in the context of follicle activation studies (Nagamatsu et al., 2019). This collagenase treatment reduced collagen in a time dependent manner such that the collagen content of reproductively old ovaries was the same as those in young counterparts following 1 h of exposure (Figure S1c‐d). Importantly, post‐treated ovaries still maintained their tissue architecture as well as the levels of other ovarian ECM components, such as HA (Figure S1e‐f).

When instrumental indentation was performed on ovaries from reproductively young and old mice as well as those from old mice treated, *ex vivo*, with collagenase, reproductively old ovaries were stiffer than young ones (young 1.98 ± 0.42 kPa; old 4.36 ± 1.24 kPa, *p* < 0.05; Figure 1d) as expected. However, *ex vivo* collagenase treatment of ovaries from reproductively old mice changed their biomechanical properties so that they were more similar to young controls, thereby rescuing the aging phenotype (2.28 ± 0.61 kPa, ns, Figure 1d). We also confirmed the efficacy of collagenase treatment in these indented samples histologically (Figure 1e‐f). Thus, reducing collagen in ovaries from mice of advanced reproductive age can fully restore the softer tissue environment to that of reproductively young mouse ovaries.

### 2.2 Ovarian stromal HA decreases with age

**Figure 2:**
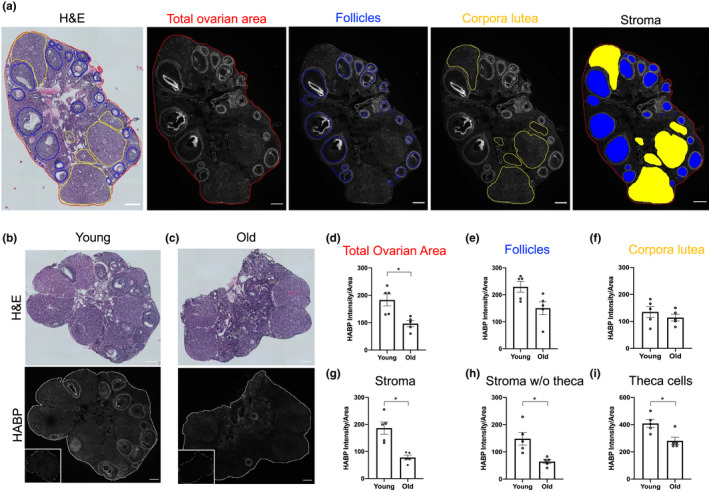
HA content decreases in the ovarian stroma and theca layer with advanced reproductive age. (a) Reproductively young mouse ovarian serial sections stained with H&E to identify ovarian structures and with the HABP assay (HA in white) to analyze total ovarian HA intensity in the follicles, in the CL, and in the stroma. (b,c) H&E and HABP assay images from ovarian tissue sections of reproductively young (b) and old (c) mice. Insets are sequential ovarian sections treated with hyaluronidase as negative controls. (d‐g) Quantification of HA intensity in the total ovarian area, follicles, CL, and stroma. Graphs showing HA intensity per area in the stroma without theca cells (h) and in the theca cell layer (i). N = 5 reproductively young and N = 5 reproductively old mice. Asterisks indicate significant differences (*p* < 0.05). Scale bar=100 μm.

In addition to collagen, the mouse ovary contains other ECM components, including HA, which may influence tissue biomechanics. HA is a well‐documented ovarian glycosaminoglycan and a critical component of the cumulus oocyte complex (COC) which develops prior to ovulation; COC HA forms a muco‐elastic matrix (Chen et al., 2016). Moreover, HA promotes tissue hydration, which is associated with softer microenvironments, and thus is an important candidate for modulating ovarian stiffness (Monslow et al., 2015; Toole, 2004). We first examined HA localization in ovarian tissue sections from reproductively young mice using a Hyaluronan Binding Protein (HABP) assay, and sequential sections were stained with H&E to visualize ovarian architecture (Figure 2 and Figure S2) (Rowley, et al., 2020). HA localized throughout the ovary in all major subcompartments, including around the endothelial cells of the blood vessels, throughout the stroma, and in and around follicles in a stage‐dependent manner (Figure S2a‐h). HA localization within primordial follicles was minimal (Figure S2d) but was observed at the primary follicle stage in the granulosa cell layer (Figure S2e). HA was prominent in secondary follicles between granulosa cells and immediately surrounding the follicle and this distribution was maintained through the antral follicle stage (Figure S2g‐h). We also observed HA in the COC and in antral cavities, as previously described (Figure S2h) (Salustri et al., 2019). HA was minimal in the corpus luteum (CL) (Figure S2i). Quantification of total HA intensity per area across follicle stages following high resolution microscopy further confirmed that the primary to secondary follicle transition is when a major increase in HA occurs both within the follicle but also in the region immediately surrounding the follicle which corresponds morphologically to the theca layer (Figure S3).

To examine whether there were age‐dependent differences in ovarian HA, we performed the HABP assay on histological sections from reproductively young and old mice and calculated HA intensity per area for the total ovary and distinct subcompartments, including follicles, CL, and the stroma (Figure 2a‐c). HA was reduced in the total ovarian area with age (*p* < 0.05), which was due to a specific reduction in the stromal compartment (*p* < 0.05; Figure 2d‐g). When performing the analysis of the stroma, HA in the theca layer was considered part of the stroma since these cells are derived from endogenous ovarian cells and from the mesonephros (Liu et al., 2015) (Figure S4a‐b). However, the theca layer is associated with follicles, which decrease in number with age. Thus, to rule out the possibility that reduction of stromal HA with age was an artifact of reduced follicle numbers, we re‐analyzed the data with theca‐associated HA analyzed as part of the follicle instead of the stroma (Figure S4c). With this method, the total HA intensity per area in the stroma of reproductively old mice was still reduced compared to young counterparts (*p* < 0.05; Figure 2h). In addition, HA intensity was also reduced in the theca cell layer in ovaries from reproductively old mice compared to young controls (*p* < 0.05; Figure 2i and Figure S4d‐e). These data demonstrate that age‐associated HA reduction is a stroma intrinsic phenomenon and not dependent on follicle number.

### 2.3 Expression of enzymes involved in HA synthesis and degradation is dysregulated with age in the ovarian stroma

**Figure 3:**
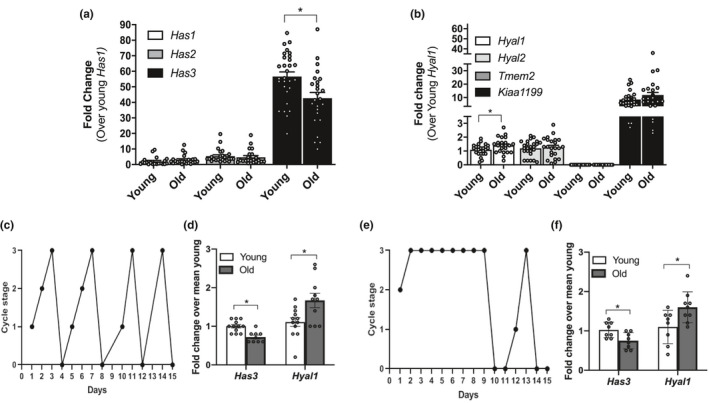
Ovarian stromal *Has3* and *Hyal1* undergo age‐dependent transcriptional changes independent of estrous cyclicity. Relative gene expression levels of (a) hyaluronan synthases and (b) hyaluronidases in ovarian stroma from reproductively young and old mice. The data are shown as a fold change expression over *Has1* (a) or *Hyal1* (b) in reproductively young mice. (c) A representative plot of a mouse exhibiting normal estrous cyclicity over a 15‐day period. (d) Graph showing the relative expression of *Has3* and *Hyal1* in reproductively young and old mice with normal estrous cycles. (e) A representative plot of a mouse exhibiting abnormal estrous cyclicity over a 15‐day period. (f) Relative expression of *Has3* and *Hyal1* in reproductively young and old mice with abnormal estrous cycles. (c and e) 0‐3 represent the different cycle stages: diestrus (0), proestrus (1), estrus (2), and metestrus (3). N = 30 stromal samples from reproductively young mice; N = 29 stromal samples from reproductively old mice. Asterisks indicate significant differences (*p* < 0.05)

To investigate the mechanism underlying HA reduction with age, we profiled the expression of hyaluronan synthases (*Has1*, *Has2* and *Has3)* and hyaluronidases (*Hyal1*, *Hyal2*, *Tmem2* and *Kiaa1199*) using RT‐PCR on stromal‐enriched ovarian tissue from which oocytes and granulosa cells had been removed (Tingen et al., 2011). *Has3* and *Kiaa1199* enzymes were the most abundantly expressed hyaluronan synthase and hyaluronidase, respectively, in the ovarian stroma irrespective of age (Figure 3a‐b). Of the enzymes examined, *Has3* and *Hyal1* were the only ones that exhibited an age‐dependent change in expression, with *Has3* expression decreasing with age and *Hyal1* expression increasing (*p* < 0.05; Figure 3a‐b). Importantly, the changes in *Has3* and *Hyal1* expression persisted even when we stratified the results based on whether mice had normal or abnormal estrous cycles, demonstrating that the observed gene expression differences were primarily age‐dependent (Figure 3c‐f). *In situ* hybridization demonstrated that both *Has3* and *Hyal1* mRNA localized to the ovarian stroma and validated that the expression of these transcripts decreased with age in this subcompartment (Figure S5a‐b). In addition, *Has3* and *Hyal1* transcripts were expressed in granulosa and theca cells and *Has3* transcripts in oocytes (Figure S5a‐b).

### 2.4 HA polydispersity does not change in the whole ovary with advanced reproductive age

**Figure 4:**
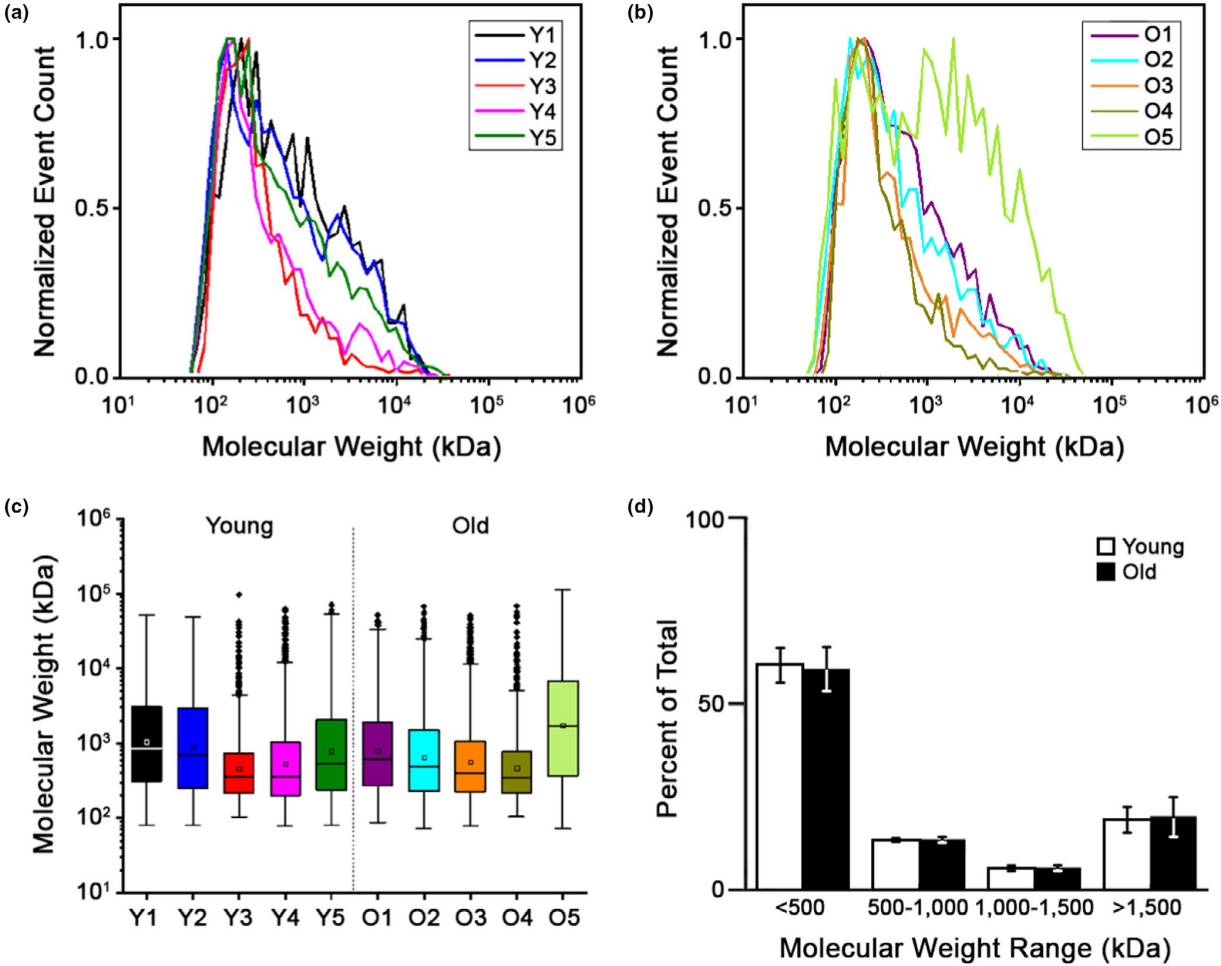
LMW‐HA fragments do not accumulate in the aging ovary. MW distributions of total HA isolated from reproductively young (a) and old (b) mouse ovaries. (c) Box plots showing the mean HA MW for each individual ovary studied. (d) Plot comparing the allocation of total ovarian HA in the MW ranges of <500, 500‐1000, 1000‐1500, and >1500 kDa, respectively, between reproductively young and old mice. N = 5 reproductively young, N = 5 reproductively old ovaries

Decreased *Has3* and increased *Hyal1* expression in the ovarian stroma with age may be indicative of reduced synthesis coupled to increased fragmentation of HMW‐HA into LMW‐HA, contributing to ovarian HA loss with age. We previously demonstrated that LMW‐HA fragments can trigger a Th2 inflammatory response in ovarian stromal cells which is predicted to increase fibrogenic pathways (Rowley, et al., 2020). Thus, we evaluated HA mass distribution in individual ovaries from reproductively young and old mice using solid‐state nanopore sensor technology (Rivas et al., 2018). We employed an optimized protocol to extract HA from tissues, the MW fidelity of which was confirmed through comparative analysis of model polydisperse HA (*Streptococcus zooepidemicus*) before and after isolation (Figure S6a). The mass distribution of ovarian HA was similar between age cohorts, ranging from: 63 ‐ 38477 kDa (mean: 373.2 ± 126.5 kDa) in reproductively young mice and 59 ‐ 44281 kDa (452.8 ± 317.3 kDa) in reproductively old mice (Figure 4a‐c and Table S1). We then evaluated the percent distribution of ovarian HA in specific MW ranges, including <500 kDa, 500‐1000 kDa, 1000‐1500 kDa and >1500 kDa (Figure 4d, Table S2). The majority of HA in the ovary was <500 kDa, but there were no age‐dependent differences across MW classes (Figure 4d, Table S2). There was also no obvious relationship between the MW distribution of HA and the estrous cycle stage of each animal (Figure 4c, Figure S6b). Thus, although aging is associated with a total decline of ovarian HA, the mass distribution remains constant at least at the level of the whole organ.

### 2.5 HA loss with age is associated with increased ovarian stiffness

**Figure 5:**
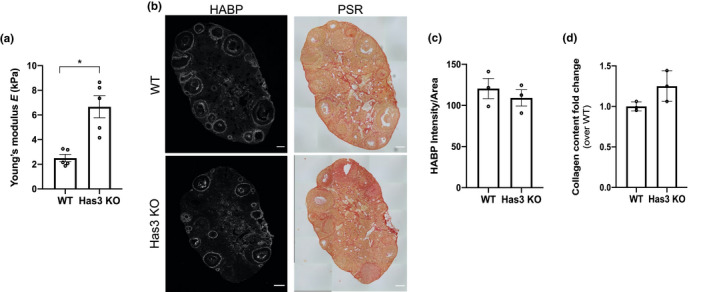
HA dysregulation is associated with increased ovarian stiffness. (a) Ovarian stiffness of WT (N = 5) and Has3 KO (N = 5) mouse ovaries. (b) Representative images of WT and Has3 KO ovaries stained with HABP (HA in white) and PSR (collagen in red). Scale bar=100 μm. (c,d) HA and collagen quantification in WT (N = 3) and Has3 KO (N = 3) mouse ovaries (contralateral to the nanoindented ovaries). HA is represented as intensity per area and collagen as fold change over WT. The asterisk indicates statistical significance

Because we did not observe age‐associated differences in mean ovarian HA mass or polydispersity, we explored the possibility that total HA reduction with age regulates ovarian biomechanical properties. To do this, we evaluated ovarian stiffness in mice that express a catalytically inactive form of the HAS3 enzyme (Has3 KO) as this was the only HA synthesis enzyme whose expression decreased in ovaries with age (Figure 3a) (Bai et al., 2005). Ovaries from age‐matched WT and Has3 KO mice were used for instrumental indentation analysis. Has3 KO ovaries were 2.7‐fold stiffer than WT mice (Has3 KO 6.67 ± 2.00 kPa; WT 2.51 ± 0.66 kPa; *p* < 0.05) (Figure 5a). HA and collagen were evaluated in the contralateral ovaries not used for instrumental indentation; there was a trend toward decreased HA and increased collagen in Has3 KO ovaries compared to WT (Figure 5b‐d). These results suggest that HA matrix, in addition to collagen, regulates ovarian stiffness and undergoes significant age‐dependent changes.

### 2.6 The age‐associated increase in collagen and decrease in HA occur in the human ovary

**Figure 6:**
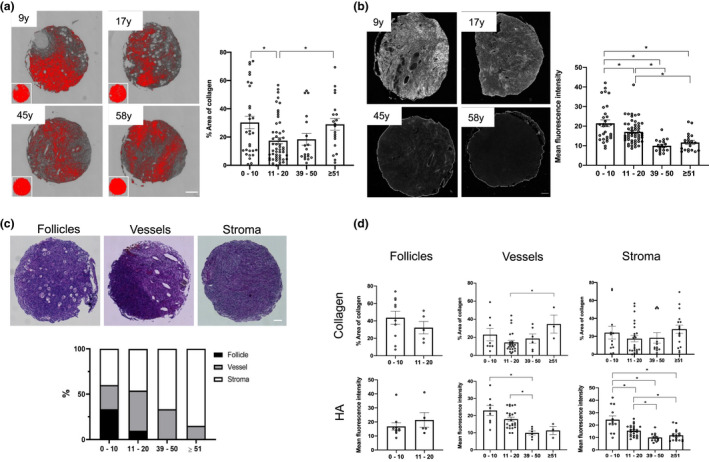
Age‐associated collagen and HA changes are conserved in humans. (a) On the left, representative processed PSR‐stained and color thresholded images for selected participants ranging from youngest to oldest cohort are shown. Scale bar=100 μm. N = 120. Graph showing the percent fibrotic area for each cohort. (b) Representative images of HABP stained TMA samples for selected participants in each of the 4 age cohorts. Scale bar=100 μm. N = 120. On the right, the mean fluorescence intensity HA is represented for each age cohort. (c) Representative H&E‐stained images containing follicles, vessels, or stroma, and the percentage of category within each given cohort is represented below. Scale bar=100 μm. (d) Graphs showing collagen and HA quantification in follicle, vessel, and stroma compartments. Asterisks indicate significant differences (*p *< 0.05).

To determine the translational relevance of our findings, we assessed whether the changes that occur with age in ovarian collagen and HA in the mouse also occur in humans. To do this, we generated a human ovarian tissue microarray (TMA) using tissue from females in 4 different age cohorts (0–10, 11–20, 39–50, ≥51 years) (Table S3). Collagen and HA content were then analyzed by staining serial histologic TMA sections with PSR and HABP, respectively (Figure 6a‐b and Figure S7a‐b). Collagen content significantly increased between the 11–20 and ≥51 age cohorts (*p* < 0.05). Interestingly, we observed that collagen levels in the youngest cohort are comparable to the oldest one, likely due to the predominance of collagen‐producing fibroblasts in the prepubertal ovary (Figure 6a). We then analyzed HA in the human ovary. HA was ubiquitously distributed in human ovarian tissue, including the vasculature, the stroma, and follicular subcompartments, as we observed in the mouse ovary (Figure 6b, Figure S7c). We also validated that the staining was specific because the signal was absent in ovarian samples pre‐treated with hyaluronidase (Figure S7c). When HA intensity was analyzed across the aging continuum, we observed a significant reduction of HA fluorescence intensity with age (Figure 6b). Although the tissue cores for the TMA array were all from the ovarian cortex, there was substantial tissue heterogeneity with varying degrees of follicles, vasculature, and stroma in each core (Figure 6c). In fact, the composition of the ovarian cortex varies with age, with cores from the youngest cohort containing follicles, vessels and stroma and those from the oldest cohort containing primarily stroma (Figure 6c). To investigate whether collagen and HA dynamics we observed with age in the human ovary depend on the subcompartment, we stratified our results by structural category. Collagen content in cores primarily with blood vessels showed a significant increase with age (*p* < 0.05), and there was a similar positive trend in the cores enriched in stroma (ns). HA intensity was significantly reduced in cores with these same structures (*p* < 0.05; Figure 6d). No differences were observed in cores with follicles, but this analysis was only possible in the two youngest age categories because follicles were absent at later ages (Figure 6d). Therefore, parallel changes in collagen and HA content occur in the ovarian stroma with age in both mice and humans.

## 3 DISCUSSION

In this study, we analyzed ovarian biomechanics in the mouse and demonstrated that ovarian tissue stiffness increased with age. An increase in collagen was in part responsible for this ovarian stiffness because, *ex vivo*, enzyme‐mediated collagen depletion in ovaries from reproductively old mice rejuvenated their biomechanical properties and reestablished the biomechanical profile of young ovaries. Although we focused on how the increase in collagen content with age influences ovarian stiffness, other aspects of the ovarian collagen matrix such as its organization, cross‐linking, and relationship to other ECM components may be relevant to aging. We also demonstrated that HA, which under physiologic conditions establishes a homeostatic matrix, regulates the biomechanical properties of the ovary because ovaries from a genetic mouse model in which a non‐functional version of the HAS3 enzyme was expressed were significantly stiffer than age‐matched WT controls. Interestingly, ovarian HA decreased with age in the stroma and theca cells possibly due to an age‐dependent shift in the expression of genes that regulate HA production, suggesting an increase in HA degradation and decrease in its synthesis. These findings demonstrate that dysregulation of two ECM components—collagen and HA—are responsible for changes in the stroma which dictate biomechanical properties of the ovarian microenvironment. Importantly, we validated that age‐associated changes in the ovarian collagen and HA matrices observed in the mouse model also occur in human ovarian tissue, underscoring translational relevance.

In the ovary, HA is often considered with respect to COCs, which expand at the time of ovulation and are uniquely soft and elastic due to their HA content (Chen et al., 2016). The current study has greatly broadened our understanding of HA in the mouse and human ovary. HA localizes to the vasculature, stroma, and follicles in the mouse and human ovary, and in contrast to the HA‐enriched COC, the aging ovarian stroma is an HA depleted and stiff environment. Future studies are needed and ongoing to determine the precise relationship between stiffness, HA, and fertility using the Has3 KO mice at different ages. Our expectation is that stiffness would be further magnified with age in reproductively old Has3 KO mice. Importantly, data from the naked mole rat (NMR) clearly link HA to organismal aging and potentially to reproductive longevity. The NMR is a rodent with extreme lifespan and healthspan of greater than three decades (Tian et al., 2013). Key to longevity in the NMR is production of large amounts of HA, which protects against damage due to ROS, elicits robust stress‐mediated protective signaling, and enhances early contact inhibition (Tian et al., 2013). Interestingly, NMR queens can reproduce throughout their entire lifespan, suggesting an important relationship between HA levels and reproductive function. This concept is further supported by the observation that mice deficient in the expression of a hyaluronidase (*Hyal1*) have extended reproductive function (Dumaresq‐Doiron et al., 2012).

The physical environment of the ovary regulates normal follicle development, and it changes with conditions such as polycystic ovarian syndrome in which mechanically regulated ovarian signaling pathways are altered (Hornick et al., 2012; Shah et al., 2018; Wood et al., 2015). Mechanical stress can also impact follicle activation, with compression and hyperbaric pressure maintaining follicle quiescence (Nagamatsu et al., 2019). In addition, *in vitro* studies of follicles grown in alginate hydrogels of different percentages, and thus stiffnesses, demonstrate that a stiffer environment impairs follicle growth, alters the androgen‐to‐estrogen ratio, and results in the production of gametes with decreased meiotic potential (Jiao & Woodruff, 2013; Tagler et al., 2014; West et al., 2007; Xu et al., 2006). Interestingly, we have noted that follicle growth dynamics are significantly altered with age, with smaller diameter follicles for any given oocyte diameter in reproductively old mice compared to young controls (Duncan et al., 2017). Knowing the exact Young's modulus of reproductively young and old ovaries will enable *ex vivo* studies which more precisely mimic the aging environment to determine the effect micromechanical stiffness has on specific ovarian subcompartments. Beyond impacting follicle development and quality, a stiffer microenvironment may also provide a physical barrier to other aspects of ovarian function. For example, ovulation involves a continuous cycle of follicle rupture and wound repair, and a stiff environment may impede these dynamics. In fact, we recently noted a significant age‐associated increase in failed ovulation following hyperstimulation (Mara et al., 2020).

The age‐associated changes we observed in ovarian HA may have functional consequences that transcend a biomechanical role. In this study, we demonstrated that HA undergoes a dramatic organization around follicles between the primary and secondary stages. These developmental stages mark an important biological transition when preantral follicles begin to become gonadotropin dependent, develop a theca layer, and are thus able to convert androgens into estrogens (Richards et al., 2018). HA increases during this transition specifically at the region of the theca layer, which is consistent with findings in other rodent species (Hatzirodos et al., 2012; Takahashi et al., 2014). These observations raise the possibility that HA may be involved in theca cell function. In support of this idea, we observed an age‐associated decrease in HA in the theca layer, and it is known that androgen levels decline with age indicative of compromised theca cell function (Davison et al., 2005).

In this study, we used two distinct methods to evaluate HA. Because HA is non‐immunogenic, the field relies on indirect methods to detect this molecule. We used an optimized HABP assay to visualize HA in the ovary and noted an age‐associated decrease in overall ovarian HA content (Rowley, et al., 2020). However, because the HABP assay is indirect, we cannot exclude the possibility that these findings instead reflect age‐related changes in hyaldherins, or hyaluronan binding proteins, which may impede binding of the biotinylated HABP we use to localize ovarian HA (Toole, 2004). Hyaladherins influence HA signaling and overall ECM structure, and thus analysis of how their composition and function change in the ovary with age represents an important frontier for future research. In addition to examining overall content, we also quantified HA polydispersity using solid‐state nanopore technology (Rivas et al., 2018). This is the first time nanopore technology has been applied to HA analysis in a biological tissue under physiologic aging conditions. We did not observe a difference in the mass distribution of HA between individual ovaries from reproductively young and old mice. This is a surprising result given that HMW‐HA can be fragmented into LMW species by hyaluronidases or exposure to ROS, both of which increase with age in the ovary (Lim & Luderer, 2011). However, our analysis was done on whole ovaries, so we do not know what is happening within specific ovarian subcompartments. We cannot exclude the possibility that there is a localized increase and accumulation in HA fragmentation. In addition, since we are only examining two timepoints (reproductively young and old), we do not know the dynamics of HA fragmentation across an aging continuum. Perhaps there are regional differences in polydispersity or a temporal increase in HA fragmentation that is short‐lived and earlier in the aging process than we examined here but sufficient to trigger an inflammatory response. In support of this hypothesis, we recently demonstrated that LMW‐HA fragments induce an inflammatory response in isolated stromal cells *in vitro* similar to that observed in the ovary with advanced reproductive age (Rowley, et al., 2020), suggesting a multi‐factorial role of HA in ovarian aging. Moreover, these same fragments compromise hormone production and gamete quality in an *in vitro* follicle growth model (Rowley, et al., 2020). Thus, future studies to investigate HA mass profiles in specific ovarian compartments are warranted.

This work provides novel insights into how the biomechanical properties of the ovarian microenvironment change with age and provide mechanistic insights into which ECM components are primary regulators of ovarian stiffness. Our data suggest that appropriate levels and dynamics of both collagen and HA matrices are essential to create a homeostatic ovarian environment. The conservation of these age‐associated changes in collagen and HA in human ovarian tissue suggests that therapeutic strategies which alleviate tissue stiffness, such as anti‐fibrotic or pro‐hyaluronan agents, may improve reproductive longevity and ultimately general health, since the age‐dependent decline in gonadal hormones contributes to global aging phenotypes in women. Furthermore, these strategies extend beyond reproductive aging to conditions such as polycystic ovarian syndrome, which is also characterized by increased ovarian stiffness and reduced gamete quality (Dumesic & Abbott, 2008; Wood et al., 2015; Wood et al., 2007).

## 4 EXPERIMENTAL PROCEDURES

### 4.1 Animals

Reproductively young CB6F1 mice (6–12 weeks old) were obtained from Envigo (Indianapolis, IN) and reproductively old CB6F1 mice (14–17 months old) were obtained from the National Institutes on Aging Aged Rodent Colony (National Institutes of Health, Bethesda, MD). Based on a linear extrapolation of age, the 14‐17 month old cohort corresponds to women in their late thirties to early forties (Duncan et al., 2017; Pan et al., 2008). Mice were housed at Northwestern University's Center for Comparative Medicine under constant temperature, humidity, and light (14 h light/10 h dark) with free access to food and water. All animal experiments described here were approved by the Institutional Animal Care and Use Committee (Northwestern University) and were performed in accordance with National Institutes of Health Guidelines. For the genetic perturbation studies, the Has3 KO mice were on a C57BL/6 J background and compared to age‐matched WT mice derived from the Has3 KO strain (after more than 10 backcrosses) and bred in parallel. These mice were originally obtained from Dr. de la Motte and Dr. Kessler at Cleveland Clinic and were bred at the University of Kansas Medical Center (KUMC) in accordance to protocols approved by the KUMC’s Institutional Animal Care and Use Committee. These mice were generated by creating a mutation in the genomic DNA encoding for the catalytic domain of the HAS3 enzyme, producing a catalytically dead enzyme (Bai et al., 2005). The mice were kept in a 10 h light/14 h dark cycle with free access to food and water and were used at 5‐months of age (mature adult).

### 4.2 Human ovarian tissue samples

Fixed paraffin embedded specimens of human ovarian tissue were obtained through two IRB‐approved tissue repositories following informed consent: the Oncofertility Consortium's National Physicians Cooperative (OC‐NPC) and the Northwestern University Reproductive Tissue Library (NU‐RTL). Samples from sixty participants were chosen for this study and consisted of four age cohorts: 0‐10, 11‐20, 39‐50, ≥51 years. We excluded samples from participants with diagnoses that may directly or indirectly affect the ovary (e.g., leukemia, ovarian cysts, uterine sarcoma, BRCA2+, pelvic mass, ovarian cancer, other ovarian tumors, endometriosis, polycystic ovarian syndrome, inflammatory/autoimmune conditions, or other possible confounders of fibrosis), with a history of prior chemotherapy and/or radiation, or with prior treatment with hydroxyurea.

### 4.3 Human ovarian tissue microarray (TMA)

To generate the TMA, cortical areas suitable for coring were defined and marked by a certified gynecologic pathologist (Dr. J.J. Wei) using an H&E‐stained 5 μm thick section corresponding to each tissue block from each of the 60 participants described above. The Northwestern Pathology Core Facility generated the human ovarian TMA using the donor blocks and the matched marked H&E slides as a guide. Selected tissue cores (1 mm diameter and 4 mm average depth) were taken from two pre‐defined locations within the ovarian cortex using a semi‐automated Veridiam Tissue Microarrayer VTA‐100 (Veridiam Tissue Arrayer Oceanside, CA). One TMA block was constructed, containing all 60 samples in duplicate, giving a total of 120 experimental samples (Figure S7, Table S3). Controls included normal human liver, fibrotic human liver, and human ovarian cancer tissue (Table S3). Sections of 4 µm thickness were cut from the TMA block and mounted on slides.

### 4.4 Instrumental indentation and elastic modulus analysis

For instrumental indentation measurements, mouse ovaries were removed from the bursa and placed in Leibovitz's L‐15 medium (Life Technologies) supplemented with 3 mg/ml polyvinylpyrrolidone (Sigma‐Aldrich) and 0.5% Pen Strep (Thermo Fisher Scientific—L15 PVP). Prior to indentation measurements, each ovary was attached to a petri dish using tissue glue (PELCO® Pro CA44 Tissue Adhesive, Fresno, CA) and covered with 1X warm PBS. Elastic modulus (Young's modulus, *E*) of the ovaries was measured using a Bruker Hysitron BioSoft Indenter (Bruker, Billerica, MA) at room temperature with a 200 µm diameter sapphire probe. For all measurements, the loading and unloading were kept at 5 µm/s and the indenter was held at constant load for 5 seconds before unloading. A minimum of 15 unique measurements across the ovarian surface were performed for each ovary. The data were analyzed using the Hertizan contact model (OriginPro 9.2) to calculate *E*.

Ovaries from Has3 KO mice and WT controls were harvested at the University of Kansas Medical Center. From each mouse, one ovary was shipped to Northwestern University overnight at 4°C with L15 PVP to perform indentation. Overnight transportation did not affect ovarian stiffness compared to non‐transported control samples (transportation 1.71 ± 0.09 kPa; no transportation 1.55 ± 0.11 kPa; ns). The contralateral ovaries were fixed with Carnoy's Solution (RICCA Chemical Company, Arlington, TX) to perform histological analysis. The ovaries for instrumental indentation were washed with warm L15 PVP and incubated at 37°C 1 h to recover the *in vivo* temperature when they were delivered at Northwestern University.

### 4.5 Collagenase digestion and downstream fibrosis and elastic modulus analysis

For collagenase functional assays, ovaries were harvested from reproductively young or old mice (n = 1 mouse each age; Figure S1). Using 22G insulin syringes, 4 holes were created on each ovary. Old ovaries were then placed in maintenance media (MM) containing 0.58 mg/ml (118.9 U/ml) of collagenase, Type IV (Fisher Scientific) for 15 min or 1 h at 37°C. One of the ovaries from the reproductively young mouse was directly fixed with MD, and the second one was incubated in collagenase‐free MM for 1 h at 37°C. After each treatment, the ovaries were washed with MM and fixed with MD for 3 h at RT and 4°C O/N. After fixation, all ovaries were dehydrated in 70% ethanol and embedded in paraffin, sectioned (5 μm thickness), and stained with H&E and PSR as described below. The PSR‐positive area was analyzed in 10 different sections of the same ovary. To measure the impact of collagenase treatment in ovaries from old mice on elastic modulus (*E*), ovaries from 2 reproductively young mice, 4 reproductively old control mice, and 4 reproductively old mice treated with collagenase were used (Figure 1). Control ovaries from reproductively young mice were poked 4 times and incubated in collagenase‐free MM for 1 h at 37°C. Ovaries from reproductively old mice were poked 4 times each and either incubated in collagenase‐free MM, or in collagenase, Type IV‐containing MM for 1 h at 37°C. After collagenase incubation, all ovaries were subjected to instrumental indentation, fixed in MD, embedded and stained with H&E, for histological assessment, PSR to quantify collagen content and HABP for HA content.

### 4.6 Histologic staining

For H&E and PSR staining, standard protocols were used (Briley et al., 2016). The H&E‐ and PSR‐stained sections were imaged with an EVOS FL Auto Imaging system at 20X for mouse samples, and with the Leica DM6B Widefield Imaging system (Leica Biosystems, Wetzlar, Germany) at 20X for the human TMA. To quantify the percentage of PSR‐positive signal, the threshold tool in Fiji was used as previously described (Briley et al., 2016), and the fold change calculated over young or WT conditions.

### 4.7 Hyaluronan binding protein assay and ovarian subcompartment quantification

To detect HA, we performed an HA‐binding protein (HABP) (biotinylated HABP, MilliporeSigma, Burlington, MA) assay on ovarian sections as previously described (Rowley, et al., 2020). Entire mouse ovarian sections were scanned at 20X when imaged with the GFP light cube using an EVOS FL Auto Imaging system. The imaging settings were kept constant for all samples after determining the threshold. For the human TMA, we used a Leica DM6B Widefield Imaging system (Leica Biosystems) with a 10X objective. As a control, we confirmed that hyaluronidase‐treated samples (HAase) did not show a positive signal and used this to help set the threshold. For confocal imaging, the same mouse sections were imaged with a Leica SP8 and the images acquired with the LAS X Life Science microscope software (Leica, Wetzlar, Germany).

HABP staining intensity analysis on mouse ovarian subcompartments was performed by defining ROIs using Fiji. H&E staining was performed on sections adjacent to those used in the HABP assay to aid in identifying follicles, CL, and stroma based on morphology. HA staining is reported as a ratio of HABP intensity per area (intensity/area) of the whole ovary, follicles, CL, and stroma. To determine HA content in the stroma, the HA intensity/area measurement for the follicles and CL was combined and then subtracted from the HA intensity/area measurement for the whole ovary. To quantify the theca cell layer HA content, the follicle HA intensity and area was measured either with or without the theca cell layer. Then, the HA intensity/area ratio was calculated for both conditions. The HA intensity/area in the theca cells was measured by subtracting HA intensity/area in the follicles without theca cells from the follicles with theca cells. Finally, the background intensity/area (measured in the hyaluronidase‐treated samples for each ovary) was subtracted. For the human TMA, the minimum threshold was set to 5 and max threshold was set to 255, based on the human ovary whole tissue section positive and negative controls (data not shown) and the mean fluorescence intensity was measured under the analyze and measure features within ImageJ.

### 4.8 Real time PCR for hyaluronan synthase and hyaluronidase gene expression in ovarian stroma

Ovarian stroma samples were prepared as previously described (Rowley, et al., 2020), homogenized in a bead homogenization system (FastPrep 24, MP Biomedical, Solon, OH) in RLT buffer (RNeasy Mini Kit, Qiagen, Hilden, Germany) containing 2‐mercaptotehanol; RNA was isolated using the RNeasy Mini Kit (Qiagen), with on‐column DNase I treatment. After elution, RNA concentration and purity were determined using a Nanodrop 1000 (Thermo Fisher Scientific). RNA was reverse transcribed to cDNA using a High Capacity cDNA Reverse Transcription Kit (Thermo Fisher Scientific). A CFX384 (Bio‐Rad, Hercules, CA) and StepOne Plus (Applied Biosystems, Foster City, CA) machines were used to perform real time polymerase chain reaction (PCR). Using the 2^−ΔΔCt^ method, relative fold changes in stromal *Has*/*Hyal* gene expression were calculated. *Has1*,*Has2*, and *Has3* TaqMan primers were designed and synthesized by Life Technologies (Carlsbad, CA) and used at 250 nM. *Hyal1*,*Hyal2*,*Tmem2*, and *Kiaa1199* are SYBR green chemistry primers and were synthesized by Integrated DNA Technologies (Coralville, IA) and used at 100 nM. *Hyal1* and *Hyal2* were designed by PrimerBank. *Hyal1* F: TTCGATGTGGTTGCCAACAAG, R: AGGTGCCCAATTCCTCTCTGT, (accession number 145966880b3); *Hyal2* F: GCAGGACTAGGTCCCATCATC, R: TTCCATGCTACCACAAAGGGT, (Primer Bank accession number: 45331201b1); *Tmem2* F: CTTTACCTTCCGGAGTGCAG, R: CCGCTGAATCCCAAAAATAC; *Kiaa1199* F: TGATGGGAGTCGAGGTCAC, R: GAGCACTATGGAATTGTCAGG. A housekeeping gene (*18S*) was used to normalize gene expression, F: ACGGAAGGGCACCACCAGGA, R: CACCACCACCCACGGAATCG.

### 4.9 Estrous cyclicity analysis

Vaginal cells were obtained daily by gentle lavage and the cells imaged in brightfield using an EVOS FL Auto Cell Imaging system (Thermo Fisher Scientific, Waltham, MA). The stage of the estrous cycle was determined by examining the cellular morphology and composition according to established criteria (McLean et al., 2012). Animals with repeating 4‐5 day cycles were classified as “cycling” and those that showed prolonged duration at any given stage were considered “non‐cycling.”

### 4.10 RNA in situ hybridization and subcompartment quantification

mRNA transcripts were detected using the RNAScope 2.5 HD Red assay and *Has3* and *Hyal1* probes (Advanced Cell Diagnostics, Hayward, CA) on an EVOS FL Auto Imaging system using a 40X objective. Sections from 5 reproductively young and 5 old mice ovaries were used to evaluate the number of *Has3* and *Hyal1* transcripts per area in the stroma. Five different regions of ovarian stroma per mouse were analyzed and the results averaged. All the reagents were provided with the RNAScope 2.5 HD Red assay kit. RNAScope experiments were performed as described (F. Wang et al., 2012).

### 4.11 HA isolation for solid‐state nanopore analysis

First, protein components were degraded by incubating individual ovaries in 1X PBS with 0.27 U proteinase K (New England Biolabs, Ipswich, MA) at RT under rocking. After 24 h, an additional aliquot of proteinase K was added and the mixture was incubated for 24 h more to fully digest the tissue. The resulting solution was removed and transferred to a phase‐lock gel tube (QuantaBio, Beverly, MA) and 150 μL of phenol:chloroform:isoamyl alcohol (24:24:1 v/v/v (Thermo Fisher Scientific, Hampton, NH)) was added and mixed before centrifuging for 25 min at 14,000 × *g*. This resulted in segregation of HA into the upper aqueous phase (along with other glycans and nucleic acids) and protein constituents and exogenous Proteinase K into the lower organic phase. The same process was repeated twice, replacing the phenol mixture with an equal volume of pure chloroform as the organic component to remove residual phenol.

Isolation of pure HA from the aqueous phase was achieved using a procedure employed previously (Rivas et al., 2018), modified here for high‐fidelity retrieval (Figure S6a). 1.5 mg streptavidin paramagnetic beads (Dynabeads M‐280, Invitrogen, Carlsbad, CA) were conjugated with 15 μg biotinylated versican G1 domain (bVG1, Echelon Biosciences, Salt Lake City, UT) and combined with the aqueous isolate. After a 24 h incubation under rocking, the mixture was placed on a magnet to pull down the beads and the supernatant was removed. The captured beads were washed three times with 1X PBS, resuspended in 70 μL of 1X PBS, and then placed on a heating block at 90°C for 15 min to denature the bVG1 and release the bound HA. The mixture was placed on a magnet to pull down the beads (with bound bVG1) and the solution containing the released HA was finally stored at −20 °C until use.

### 4.12 Solid‐state nanopore HA molecular weight distribution analysis

Solid‐state nanopore devices consisting of a single pore (6–8 nm diameter) were fabricated in a 19 nm thin, free‐standing silicon nitride membrane supported by a silicon chip (4 mm) following methods described elsewhere (Yang et al., 2011). For measurement, a device was positioned in a custom flow cell between two reservoirs of measurement buffer (6 M LiCl, 10 mM Tris, 1 mM EDTA, pH 8.0) with one reservoir also containing the sample mixture. An Ag/AgCl electrode (Sigma‐Aldrich, St. Louis MO) was introduced to each side and connected to a patch clamp amplifier (Axopatch 200b, Axon Instruments, Union City, CA) that was used to both apply a 200 mV bias across the device and to record trans‐pore ionic current at a rate of 200 kHz using a 100 kHz four‐pole Bessel filter and an additional 5 kHz low‐pass filter applied using custom software. Resistive pulses (“events”) caused by electrophoretic translocations of HA through the pore were identified as transient reductions in the ionic current >5σ in amplitude compared to RMS baseline noise and with durations in the range of 25 μs−2.5 ms. The event charge deficit (ECD), defined as the integrated area of an event, was determined for each molecular translocation and converted to MW using a calibration standard produced with synthetic, quasi‐monodisperse HA (Hyalose, Oklahoma City, OK), as described elsewhere (Rivas et al., 2018).

### 4.13 Statistical Analysis

The normality of the sample was first evaluated by visual inspection of the distribution using Q‐Q plot (quantile–quantile plot) and using the Shapiro–Wilk test. Normally distributed samples were analyzed using Student t‐test or one‐way ANOVA for comparisons between 2 groups or more than 2 groups, respectively. For non‐normal samples, we used the non‐parametric test Mann–Whitney. Variability within the experimental group is provided as standard error of mean (SEM). All analyses were performed using the statistical package in Prism 6 (GraphPad Software, La Jolla, CA).

## CONFLICTS OF INTEREST

The authors have no conflicts of interest to declare.

## **AUTHOR** **CONTRIBUTIONS**

F.E.D and M.T.P conceived the study, designed the methodology, supervised the study, provided the resources, and involved in funding acquisition; F.A.R, S.L.M, M.T.P, and F.E.D involved in project administration; F.A.R, S.L.M, Q.T, W.S.P, F.R, L.T.Z, J.E.R, C.E.V, J.E.H, G.S.S, J.J.W, M.E.P. A.R.H, M.T.E, and F.E.D involved in investigation, analysis, validation, and visualization; and F.A.R, M.T.P, and F.E.D wrote the manuscript. All authors have read and agreed to the published version of the manuscript.

## Supporting information

Figure S1.

Figure S2.

Figure S3.

Figure S4.

Figure S5.

Figure S6.

Figure S7.

Table S1.
Table S2.
Table S3.

## Data Availability

All data generated in this study are available upon request from the corresponding authors.
