# Supplementary figures and images for: Ovarian stiffness increases with age in the mammalian ovary and depends on collagen and hyaluronan matrices
Source: Aging Cell. 2020 Oct 20;19(11):e13259. doi: 10.1111/acel.13259 (PMC7681059; doi:10.1111/acel.13259)

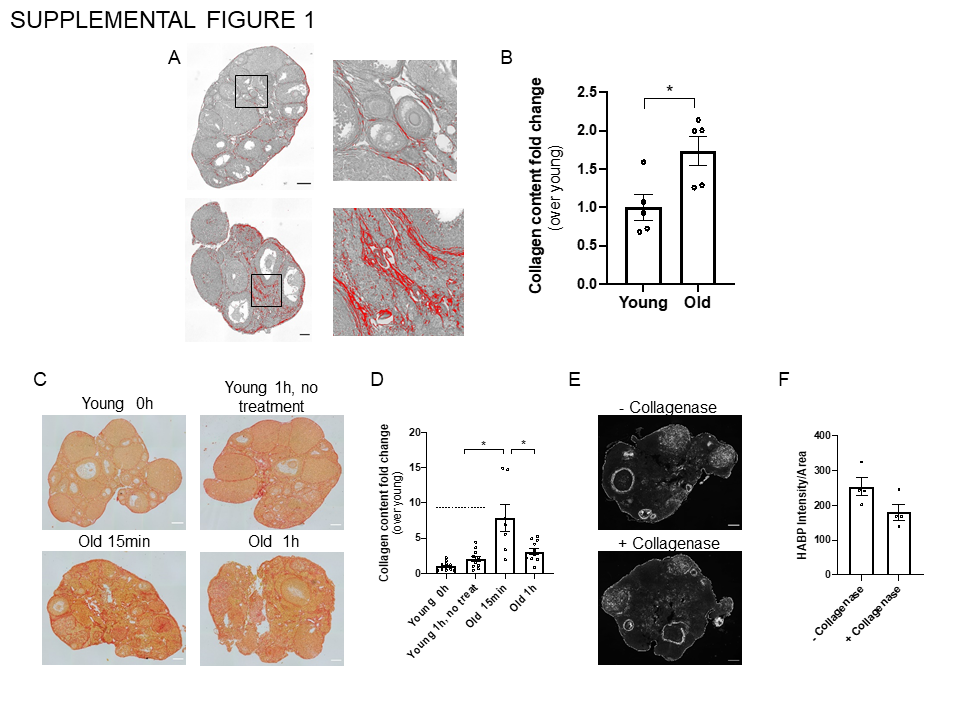

Supplement: Supplementary file 1 — Figure S1. [file ACEL-19-e13259-s001.TIF]

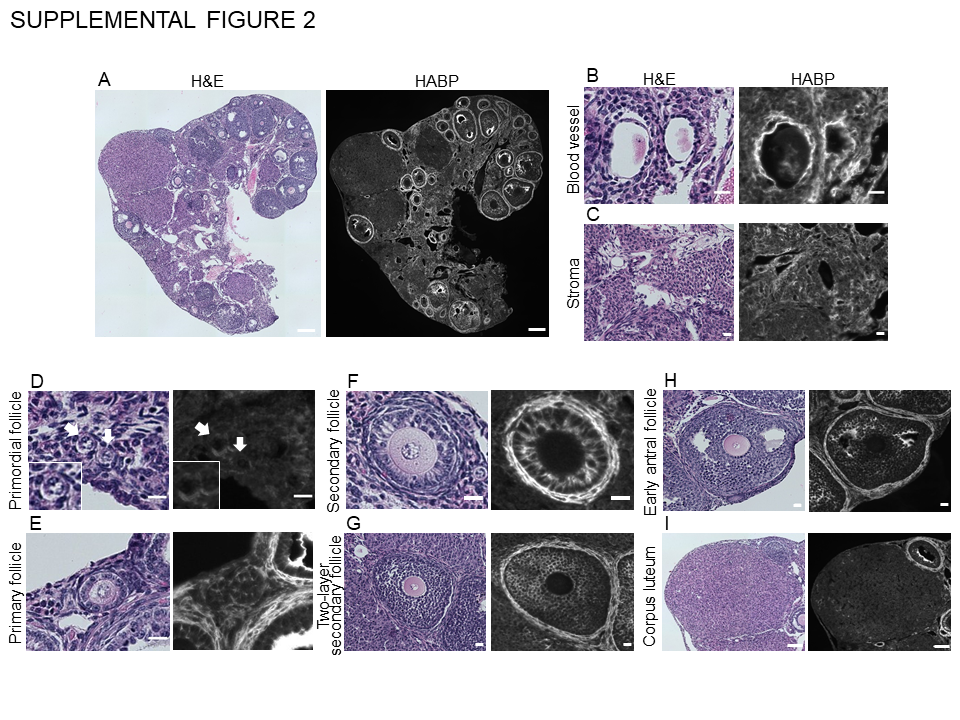

Supplement: Supplementary file 2 — Figure S2. [file ACEL-19-e13259-s002.TIF]

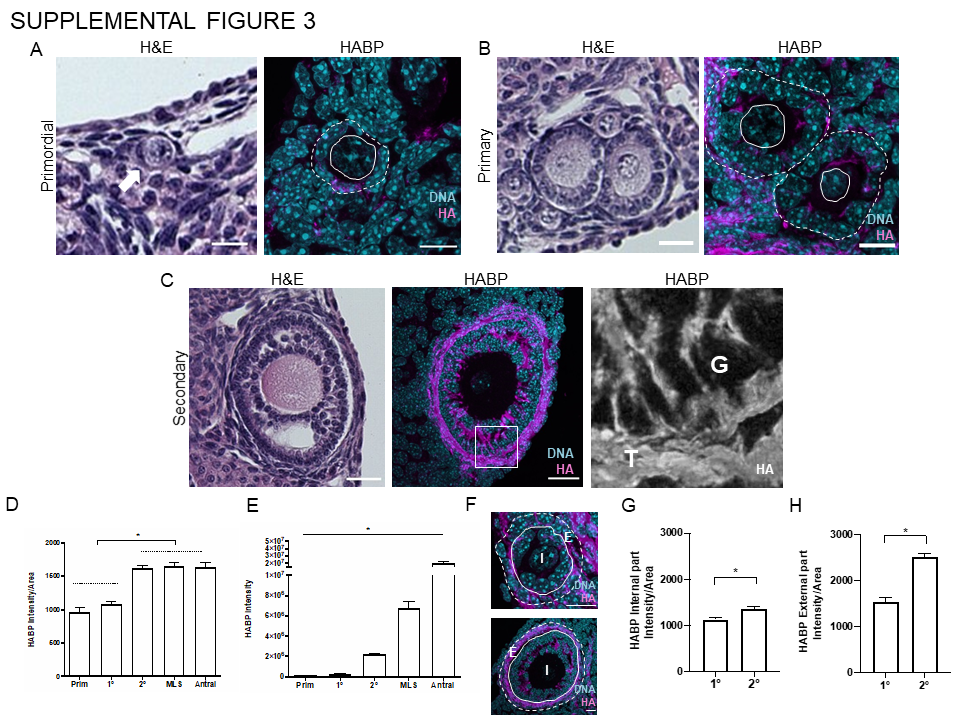

Supplement: Supplementary file 3 — Figure S3. [file ACEL-19-e13259-s003.TIF]

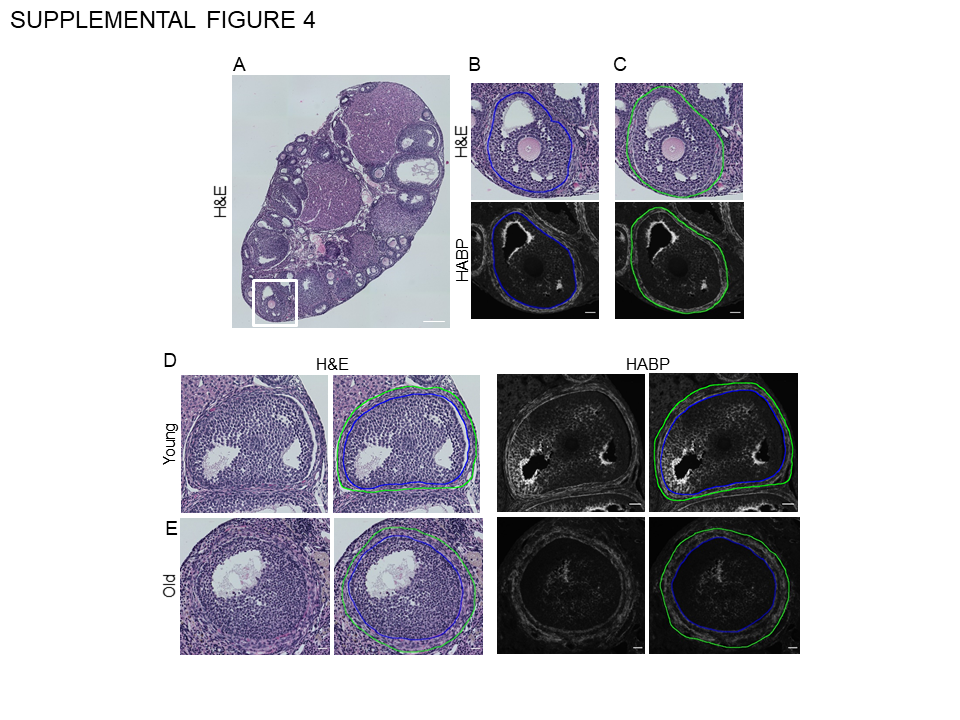

Supplement: Supplementary file 4 — Figure S4. [file ACEL-19-e13259-s004.TIF]

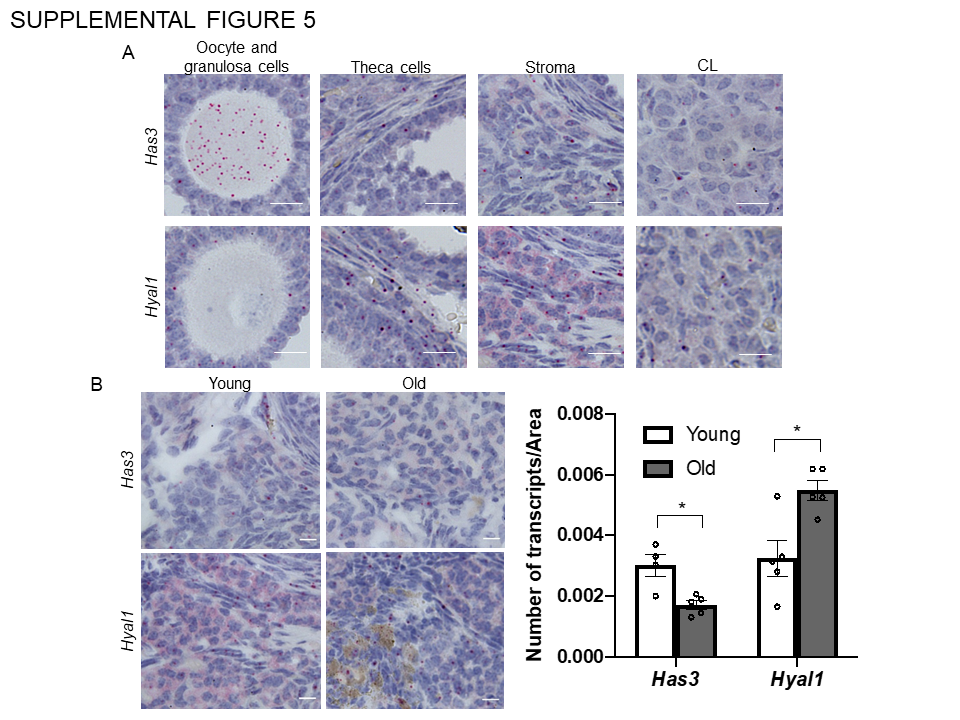

Supplement: Supplementary file 5 — Figure S5. [file ACEL-19-e13259-s005.TIF]

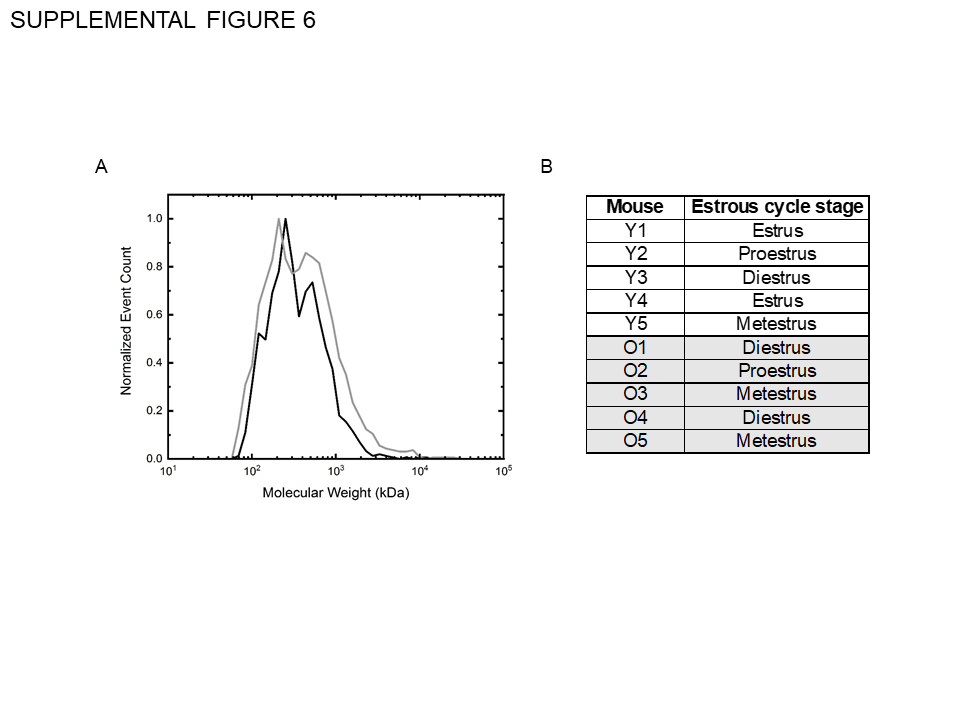

Supplement: Supplementary file 6 — Figure S6. [file ACEL-19-e13259-s006.TIF]

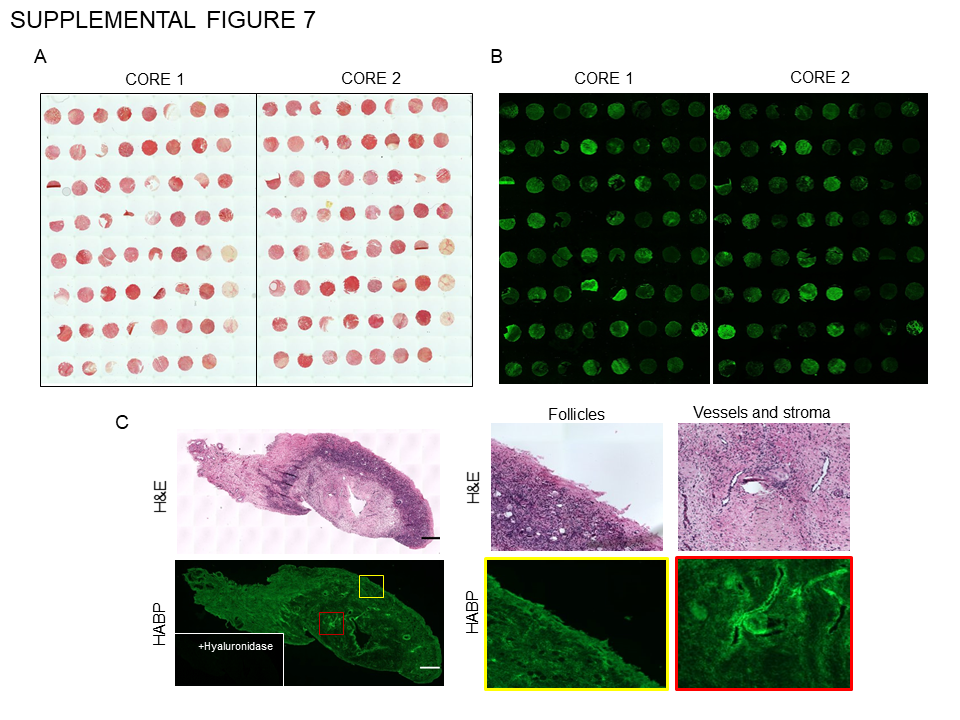

Supplement: Supplementary file 7 — Figure S7. [file ACEL-19-e13259-s007.TIF]
