# Supplementary material for: Ovarian stiffness increases with age in the mammalian ovary and depends on collagen and hyaluronan matrices
Source: Aging Cell. 2020 Oct 20;19(11):e13259. doi: 10.1111/acel.13259 (PMC7681059; doi:10.1111/acel.13259)
Supplement: Supplementary file 8 — Table S1. Table S2. Table S3. [file ACEL-19-e13259-s008.docx]

**Supplemental Figures:**

**Supplemental** **Figure 1. Collagenase treatment of reproductively old ovaries restores collagen content to those of reproductively young ovaries.** (A) Representative images of reproductively young and old ovarian sections stained with PSR (collagen in red). Scale bar = 100 μm. N=5. (B) Graph showing collagen content in reproductively young and old ovaries represented as fold change over young. (C) Representative images of PSR stained ovarian sections for each experimental condition. Scale = 100 μm. (D) Graph showing PSR positive area represented as fold change over young not treated ovaries (young, 0h). Additional groups as follows: young ovaries incubated in collagenase-free medium for 1h, at 37°C (young, 1h no treat), old ovaries treated with collagenase for 15min at 37°C (old, 15min), and old ovaries treated with collagenase for 1h at 37°C (Old,1h). (E) Representative images of reproductively old control and collagenase treated ovaries after indentation stained with HABP. Scale = 100 μm. N=4. (F) Graph showing the HABP intensity/area in old control and old collagenase treated ovaries after indentation analysis. The asterisk indicates a significant difference (p<0.05).

**Supplemental Figure 2. Hyaluronan localizes to major ovarian subcompartments.** (A) Entire ovarian section from a reproductively young mouse stained with H&E (left) and used in a HABP assay (right, white). Scale bar = 100 μm. H&E and HABP staining of ovarian vasculature, scale bar = 10 μm (B); stroma, scale bar = 10 μm (C); primordial follicles (arrows highlight two primordial follicles and one is further magnified in the inset), scale bar = 10 μm (D); primary follicle, scale bar = 10 μm (E); two-layer secondary follicle, scale bar = 10 μm (F); multi-layer secondary follicle, scale bar = 10 μm (G); early antral follicle, scale bar = 10 μm (H); corpus luteum, scale bar = 100 μm (I). Sections from N=5 ovaries were analyzed, and representative images are shown.

**Supplemental Figure 3.** **HA undergoes a prominent change in localization during the primary to secondary follicle transition**. (A-C) H&E staining and the HABP assay were performed on sequential ovarian sections from reproductively young mice. Representative images of follicles at different stages of development are shown. In each, DNA is labeled in teal, while HA is labeled in fuchsia, except for C, right panel, where HA is white. (A) Primordial follicle. Scale bar =10 μm. (B) Primary follicles. Scale bar = 10 μm. In (A-B), the dashed line outlines the follicle, and the solid line outlines the oocyte nucleus. (C) Secondary follicle; the right panel is an inset of the middle panel highlighting HA in both the granulosa and theca layers in close apposition (G=granulosa cells, T= theca layer). Scale bar = 20 μm. A total of 10 follicles per stage from 5 different mice were imaged. (D) Quantification of HABP intensity per total follicle area across the different follicles stages. (E) Graph showing the total HABP intensity per follicle. Prim: Primordial (N=23 follicles), 1°: Primary (N=40 follicles), 2°: Secondary (N=76 follicles), MLS: multilayer secondary (N=96 follicles), Antral (N=35 follicles). (F) Representative images that show the internal and external portions of primary (top) and secondary (bottom) follicles. The solid line outlines the internal part of the follicle, whereas the dashed line outlines the external part. Scale bar = 10 μm. Graph showing the HA intensity per area in the (G) internal and (H) external part of primary and secondary follicles. N=37 primary follicles and N=73 secondary follicles were analyzed. Asterisks indicate significant differences (p<0.05).

**Supplemental** **Figure 4. Strategy to quantify ovarian stromal HA, with or without the theca cell layer, to control for follicle number.** (A) H&E staining of a representative whole ovarian tissue section from a reproductively young mouse. The inset highlights an early antral follicle that is magnified in panels (B-C). (B) Early antral follicle stained with H&E and HABP assay (HA is white). Here, the theca cells are considered part of the stroma (blue line outlines the follicle excluding the theca layer). (C) H&E and HABP assay where the theca cells are considered part of the follicle (green line outlines the follicles including the theca layer). H&E and HABP stained sections of reproductively young antral follicles (D) and reproductively old antral follicles (E). The area between blue and green lines represents the theca cells. N=5 reproductively young and N=5 reproductively old mice. Scale bar A = 100 μm. Scale bar B-E = 50 μm.

**Supplemental** **Figure 5. *Has3* and *Hyal1* transcripts localize in most of the ovarian subcompartments and their expression changes with age.** (A) Representative images of *Has*3 and *Hyal1* mRNA molecules (red dots) in the oocyte and granulosa cells, theca cells, stroma, and CL in reproductively young mouse ovarian sections. Scale = 100 μm. N=5. (B) Images showing *Has*3 and *Hyal1* mRNA molecules (red dots) in the ovarian stroma of reproductively young and old ovaries. On the right, graph showing the number of *Has*3 and *Hyal1* mRNA transcripts per area in the stroma of reproductively young and old ovaries. The asterisk indicates a significant difference (p<0.05).

**Supplemental** **Figure 6. Evidence of high fidelity HA isolation methods and mouse estrous cycle analysis.** (A) Comparison of tissue-derived HA protocol to a purified *Streptococcus zooepidemicus* HA sample with known polydispersity. Graph showing the comparison of MW distributions determined in a polydisperse sample of HA before (Control PolyHA – black line) and after (Extracted PolyHA – grey line) the tissue isolation protocol. (B) Table showing estrous cycle stage of the mice whose ovaries were used in the solid-state nanopore analysis shown in Figure 4.

**Supplemental Figure 7. Human ovarian TMA array.** (A,B) PSR staining (A) and HABP assay (B) performed on sequential sections of the human ovarian TMA array. The staining was performed in duplicate (core 1 and core 2). N=120 ovarian samples. (C) Sequential human ovarian sections stained with H&E and HABP. The inset of the HABP assay shows a control section treated with hyaluronidase. Yellow and red selected ovarian areas on the HABP image are magnified on the right. The yellow square shows HABP staining on follicles located in the ovarian cortex and the red square shows HABP staining around blood vessels and in the stroma. Scale bar = 25mm.

**Supplemental Tables:**

**Supplemental Table 1: Descriptive statistics of HA distribution in individual ovaries from reproductively young and old mice. Y: Young. O: Old.**

|  | N total | Mean | Standard Deviation | Lower 95% CI of Mean | Upper 95% CI of Mean | Variance | Minimum | Median | Maximum |
| --- | --- | --- | --- | --- | --- | --- | --- | --- | --- |
| Y1 | 1,140 | 1,677 | 2,767 | 1,516 | 1,838 | 7,656,379 | 64 | 542 | 21,954 |
| Y2 | 1,994 | 1,584 | 2,711 | 1,465 | 1,703 | 7,348,353 | 64 | 454 | 20,781 |
| Y3 | 1,175 | 651 | 1,852 | 545 | 757 | 3,430,063 | 82 | 246 | 38,477 |
| Y4 | 1,898 | 943 | 2,200 | 844 | 1,042 | 4,841,877 | 63 | 250 | 26,028 |
| Y5 | 2,447 | 1,427 | 2,904 | 1,312 | 1,542 | 8,432,037 | 65 | 364 | 30,295 |
| O1 | 1,504 | 1,219 | 2,256 | 1,105 | 1,333 | 5,087,566 | 71 | 409 | 21,997 |
| O2 | 2,217 | 1,115 | 2,369 | 1,016 | 1,214 | 5,611,054 | 59 | 330 | 27,836 |
| O3 | 1,865 | 919 | 1,931 | 831 | 1,007 | 3,726,981 | 64 | 274 | 21,696 |
| O4 | 946 | 695 | 2,018 | 566 | 824 | 4,070,358 | 83 | 242 | 28,252 |
| O5 | 2,334 | 3,390 | 5,831 | 3,153 | 3,626 | 34,000,200 | 59 | 1,009 | 44,281 |

**Supplemental Table 2: Percentage (%) of total HA content per ovary with a MW of less than 500 kDa, between 500 – 1000 kDa, between 1000 – 1500 kDa and more than 1500 kDa. Y: Young. O: Old.**

| MW Range | Y1 | Y2 | Y3 | Y4 | Y5 | O1 | O2 | O3 | O4 | O5 |
| --- | --- | --- | --- | --- | --- | --- | --- | --- | --- | --- |
| <500 | 48.33 | 52.51 | 76.17 | 69.13 | 57.42 | 55.78 | 62.16 | 68.20 | 75.58 | 36.55 |
| 500<MW<1000 | 15.44 | 14.74 | 12.09 | 13.22 | 13.73 | 16.82 | 14.21 | 13.03 | 12.26 | 13.24 |
| 1000<MW<1500 | 8.42 | 6.52 | 4.09 | 4.53 | 7.36 | 7.18 | 6.27 | 4.66 | 5.07 | 8.53 |
| >1500MW | 27.81 | 26.23 | 7.66 | 13.12 | 21.50 | 20.21 | 17.37 | 14.10 | 7.08 | 41.69 |

**Supplemental Table 3: Map of the human ovarian TMA. Core 1 samples.** The map corresponds to the location of where each sample is in the TMA block core 1. Core 2 contains the same samples in a different order as it is a duplicate from core 1. AUB: abnormal uterine bleeding.
